# Ethics of AI in Medicine

**DOI:** 10.4103/singaporemedj.SMJ-2023-279

**Published:** 2024-03-26

## Abstract

We review the main ethical issues that arise in the use of AI technologies in medicine. Issues around trust, responsibility, risks of discrimination, privacy, autonomy, and potential benefits and harms are assessed. AI is a promising technology that can revolutionize, for better or worse, health care delivery. It is up to us to make it a tool for the good by ensuring ethical oversight accompanies design, development, and implementation of AI technology in clinical practice

## Introduction

Artificial intelligence (AI), including generative AI and large language models (LLMs), is revolutionizing medicine. AI assembles and connects vast amounts of information extremely rapidly to provide more effective means to achieve medical goals, such as diagnosis or treatment.

In a way, ethics is like physics. In physics, which direction an object will move is determined by the sum of the vectors of force. Each vector has a direction and strength. In ethics, the vectors are reasons for acting in certain ways. In order to determine what we should do, we should weigh the relevant reasons for different possible actions. Whether and how to employ AI is an ethical issue in this sense.

### Ethical Relativism vs Context Specificity

Ethical relativism is the view that ethics is relative to culture, time, people’s attitudes, or other factors related to particular societal, group or personal norms. Ethical relativism is arguably false. The Nazis were wrong in doing what they did, even if their culture or group endorsed those practices. The idea of universal human rights involves the rejection of ethical relativism.

However, ethics is specific to context. A type of act that is wrong in one context (killing an innocent person) may be right in another (killing an innocent person who is dying and suffering and who desires to die). The particular facts matter to ethical judgement. So it is not possible to decide whether AI is good or bad, or should or should not be employed. It will depend on the particular facts of the case and on the relevance of the different values, or reasons for actions, in different cases.

## 3 Case Examples

### 1 Breast Cancer

Researchers at the University of Cambridge recently developed a prognostic and treatment algorithm for breast cancer based on data from almost one million women.(1) Male patients were excluded from the modelling process because breast cancer is rare in men (as are clinical trials) and the cancer presents and behaves differently. The algorithm was selectively deployed on female patients. Therefore, it produces the best treatment options for female breast cancer patients. (2)

### 2 DermAssist

Google developed a skin disease algorithm in 2020 to diagnose skin conditions, including melanoma. It was as good as dermatologists and better than GPs or nurses. In 2021, Google launched the DermAssist smartphone app. It was released to patients of all skin tones in Europe and granted a CE mark as a Class I medical device in the EU, which is a form of self-certification. (3) The DermAssist app has also not received regulatory approval in the USA. (4)

Google marketed the DermAssist app as merely a search tool (a “journey”), rather than a medical diagnostic device, but various press release and website materials may have led patients to believe they could use it to make a medical diagnosis. (5) The model underlying DermAssist was also criticised for being trained and validated using data heavily skewed toward lighter-skinned patients. For Type VI skin on the Fitzpatrick scale (the darkest), only 46 samples (out of 16,530) were used for training and only 1 Type VI sample (out of 4,146) was used for validation. (6) Consequently, there are concerns about its reliability in darker skinned patients.

### 3 Algorithmic High-Risk Care Management

“High-risk management” programmes use AI to provide patient risk scores, which are then used for patient selection in ‘high-risk care management’ programmes. They have raised concerns about race-based discrimination, as Black patients were under-represented compared to White patients in such programmes across levels of sickness. (7) The reason for this outcome is that the algorithm used past medical expenditure by patients with private insurance as a proxy for health care need. However, Black patients, even with insurance, utilized services less, so the measure underestimated their true health care need. It was postulated that this lower utilization was due to obstacles to access or distrust of the health care system based on past injustices and exploitation such as the Tuskagee Syphilis experiments, where Black patients with syphilis were deliberately left untreated to follow the course of syphilis.

These examples show many of the risks and potential harms of AI in medicine: perpetuating injustice and inequality, distortion and subversion of the doctor patient relationship, undermining of autonomy and consent, and harm to patients. Importantly, we see the risks and potential harms from the use of AI in medicine appear across the therapeutic process, from pre-consultation to diagnosis to treatment selection and recommendation, and further disease management and prevention. Whether and how an AI should be pursued in a particular context in medicine will involve weighing and managing the risks and benefits. Let’s first consider the dangers of AI in medicine.

## Risks and Reasons to Regulate AI in Medicine

In this section, we outline 10 ethical risks that can (and do) arise when using AI in medicine. As mentioned, these risks span the use of AI across the therapeutic chain – from pre-diagnosis to diagnosis to treatment to disease management. These risks are not meant to be exhaustive, nor do they (or some sub-set of them) collectively form any hierarchical structure. They reflect themes and concerns from ethics that arise when using AI.

While some of these risks might be inter-related (e.g. reliability of AI systems and their ability to provide explanations can affect the extent to which we trust such systems), they have been specified in a way to capture the key elements of the literature around the ethical concerns from the use of AI in medicine. These concerns have been captured for the purposes of this commentary not through a systematic meta-analysis, but through a more subjective selection of relevant discussions as referenced throughout this paper.

### 1 Effectiveness, Reliability and Evaluation

In medicine, pharmaceuticals undergo strict regulation and require evidence from clinical trials before they are licensed. AI does not require such strict evaluation. For example, the DermAssist app is only self-certified in Europe, though this may not be permitted in 2025 as new European legislation is introduced. AI does not require randomized controlled trials. For example, AI based on time-lapse videography of embryo development has been introduced to select embryos in in vitro fertilization without any randomized controlled trials comparing to human embryologist selection. While performance data are impressive, there are a number of problems with such non-ecological evaluation, especially of non-interpretable black box models, and a number of recommendations have been made which pertain to AI in general – see Table 2. (8)

Randomized controlled trials have been the vanguard in the evidence-based medicine revolution. When it comes to AI, they should be performed on a system formed by AI and the humans who use the AI, since the result is determined both by the AI and by how it is used. This is obviously complicated, not least because it is difficult to predict the contribution of the human factor in the use of AI in clinical settings. Moreover, AI evolves quickly, so the RCT is not as adept as it is for drug trials. New forms of evaluation of AI will need to be determined to assess it in ecological environments – that is, in the context in which it is actually being deployed. This will need to be richer, deeper and more comprehensive than typical post-marketing surveillance.

### 2 Justice, Inequality, Bias, Discrimination, and Fairness

As all of the examples in the introduction show, AI will typically have different (unequal) performance for different groups. Because AI uses Big Data, there is potential for grouping individuals into many different categories. Performance of medical AI can be measured for such specific groups. The main problems arise when the criterion for such grouping is one of the “protected categories or characteristics”. These typically single out groups which have been subjected to discrimination in the past and for whom differential treatment must be explicitly and convincingly justified. In Singapore, these include: (i) age, (ii) nationality, (iii) sex, marital status, pregnancy status, caregiving responsibilities, (iv) race, religion, language, (v) disability and mental health conditions. This has raised questions in the Dermassist and High Risk Care examples. There is a question as to whether the differential treatment based on sex and skin colours, which correspond to protected characteristics, is a case of discrimination and injustice.

Discrimination occurs when like cases are treated differently without there being a significant enough morally relevant difference. What counts as significant enough difference to justify differential treatment is a value-judgement, and is up for debate. In the Breast Cancer case, there are putatively morally relevant differences between male and female cancer. The reason why they are treated differently by the algorithm is that that type of cancer is more prevalent in women, and therefore the algorithm is more effective when is trained with more data about women. Effectiveness is a morally relevant factor as it means more cases of cancer can be treated and lives saved.

Whether unequal outcomes represent injustice, and if they do represent injustice how that should be addressed, are important and difficult questions. The answers depend largely on what theory of justice one employs (see Table 2).

There are at least four responses, which are not all mutually exclusive, to unjust outcomes:

1. Correct training sets so that all groups have equal or fairer outcomes. This often called inclusion and diversity. This was proposed for the High Risk Care case.
2. Inform relevant groups of performance data (sensitivity, specificity, calibration, etc) relevant to their group and allow members to choose whether to employ the AI. This could be required in DermAssist.
3. Exclude groups for whom the AI is less effective and reliable (for instance in the Breast Cancer case)
4. Employ the AI anyway if utility for all groups is sufficiently high.

The goal of precision medicine is to make predictions and apply treatments which are specific to individuals. As AI uses groups of smaller and smaller size, which are more and more relevant to the individual (eg women aged 20-25 with a BMI of 20 and a family history of diabetes), performance will become less reliable as there are fewer and fewer members of that group whose data can be used to train the algorithm. Membership of large protected categories may be medically relevant (eg sex in breast cancer and skin pigmentation in melanoma are relevant because they are associated with significant variation in risk profile for those conditions). So the questions of inequality of outcome and sufficient performance involve value judgements, including which theory of justice to employ, for example whether suboptimal results in a particular protected category is justified by overall higher efficacy on the population considered as a whole.

There are various kinds of bias which can occur in AI design. The High Risk Care case is an example of ‘label bias’, in the sense that the data label used (‘medical expenditure’) meant different things for different patients. This was ‘because it is an imperfect proxy [for future healthcare need] that is subject to health care disparities rather than an adjudicated truth’. (9) Other biases include ‘minority bias’, which results in the underrepresentation of minority groups in the training data (as in the case of DermAssist), ‘missing data bias’, when data is missing for protected groups in a non-random fashion, and ‘informativeness bias’, when chosen features are less informative for prediction in a protected group. (10)

### 3 Privacy and Confidentiality

AI requires Big Data and this risks breaching the privacy and confidentiality of patients. There are a number of responses to these concerns.

Firstly, valid consent can be obtained for data usage. Because it is difficult to predict the different ways in which data will be used, and to what extent individual will consent to unknown future uses, different models of consent have been introduced alongside the traditional one, to better account for the possibilities opened up by Big Data. These include “broad consent”, which enables individuals to consent to a wide range of more or less specified future uses; (10) meta-consent, where individuals retain control over which type of consent they will want to give to future uses (e.g. blanket consent on certain types of uses, but specific consent on other types);(11) and dynamic consent, where consent is personalized and based on interactive online platforms where participants can constantly engage as they see fit, in real time. (12)

Secondly, data can be de-identified or anonymised to protect privacy, though there are risks of reidentification, which is linked to the level of ‘uniqueness’ of each individual. (13)

Thirdly, new authorities can be created to manage data and data linkage, which protect confidentiality, such as the TRUST platform in Singapore. Other models have been suggested, including use of novel privacy-protection technologies, such as “block chain” technologies. (14)

One method of ethics (see Box 2) is consistency across relevantly similar cases. This could imply that the standards of protection of disclosure of data in other areas of life (such as social media, purchasing, browsing, etc) should employed for the use of medical data. Whatever level of risk is acceptable in those other areas, should be considered acceptable in the case of medical data. One possible objection to this claim, however, is that medical data is more sensitive, though this may not always be the case.

### 4 Machine Paternalism and Respect for Autonomy

Paternalism involves doing what is in the someone’s best interests, against that person’s expressed preference. Soft or weak paternalism consists in doing this when someone’s decision-making capacity or information base is reduced, so that an individual is not in the position to adequately assess what is in their best interest. Hard or strong paternalism is acting in the patient’s interests without their consent when they are competent, that is, against a sufficiently competent and informed patient’s own judgment about best interest.(15) In last 20 years, there has been a move away from paternalistic medicine towards models which give greater importance to patient autonomy. Alternative models include the informative (“shared decision making”) model (doctor provides information, patient provides values), interpretative (where doctor helps the patient to identify values) and deliberative (doctor deliberates with patient). (16)

AI threatens to reintroduce paternalism by failing to consider the patient’s values. Instead, it relies on the values of those who programme AI. This is particularly the case when we consider AI systems used for treatment recommendation (rather than, for instance, diagnostics). For example, IBM’s Watson for Oncology system ranks treatments according to improvements in length (not quality) of life and does not ‘encourage doctors and patients to recognize treatment decision making as value-laden at all’. (17) This argument can be extended to any AI system that, like Watson for Oncology, prioritizes certain treatment recommendations over others, as doing so requires a metric (like longevity) to make that prioritization. By picking a value or values to be maximized, AI excludes other values which may be important to the patient. This approach can be called “machine paternalism”.

McDougall argues for value-flexible design, which involves creating AI capable of accommodating the patient’s values. (18) However, we need not go this far. As long as doctors understand the goals of AI, and its performance according to a range of relevant values, this information can be explicitly communicated to the patient. As long as doctors involve patients in dialogue about what matters to them, and relate this to available AI, the utility and limitations of AI in assisting diagnosis or treatment for the patient can be identified. For these reasons, doctors must be involved in the translation of AI recommendations into patient-centred care.

### 5 Accommodating Value Pluralism and Disagreement

AI is a tool. It must be designed to perform a task, or achieve a goal. Setting goals involves aiming at something that is of value, worth achieving. In medicine, these values are often widely agreed: prolongation of life or improvement of its quality. But, as we have seen, these can be in tension and there can be value disagreements about what is quality of life, what constitutes a good life or well-being. AI designers (especially those designing systems that go beyond diagnostics into treatment recommendation – either directly or indirectly through patient selection as in the High Risk Care Management case example) must set these values, though generative AI might create new values. But there is widespread disagreement about value, such as the use of enhancement technologies, or gender-affirming care, or love drugs to alter sexual orientation.

The necessity to set values for programming or the evaluation of AI invites the possibility of machine paternalism – the acceptance of a narrow, disputed or implicit set of values. We discuss in section below how AI can address value pluralism and disagreement.

### 6 Responsibility

Responsibility for an outcome is usually, ethically and legally, dependent on the degree of control an agent had over the outcomes and the foreseeability of the consequences. Culpable ignorance exists when an agent should have known better.

One of the great conundrums facing today’s clinicians who choose to use AI is about who is responsible if harm occurs. (19) Legally, this is a complex question which, in some ways, is yet to be decided. Equally in ethical terms, moral responsibility depends on whether a clinician exercised sufficient care in the application of the AI system. The buck generally stops with the treating clinician who takes on responsibility for the patient, unless there is fault in the technology (that is, the AI does not do what it is supposed to do), which could not have been foreseen or discovered by the treating doctor.

Responsibility will require that clinicians are able to evaluate evidence of performance and use AI appropriately, explaining its benefits, risks and their level of confidence in it for a particular patient. In this way, using AI in medical contexts is little different from using nay other medical tool, such as drugs and devices. But it will require upskilling in education about AI. It will also require doctors to understand the values driving AI, its likely outcomes, and the patient’s own values. Doctors will be as important and responsible as ever.

Blame is a function of responsibility for harm caused. Doctors will be blameworthy if they fail to satisfactorily evaluate the performance of AI, fail to communicate the risks and benefits of it, and its alternatives, and their rational confidence in its performance, or apply it inappropriately.

The rules for designers are somewhat different. They will be responsible if they fail to make clear the values which AI seeks to maximise, and the limits of confidence in performance for relevant, specific patient groups but also if it is insufficiently safe, reliable and effective.

### 7 Trust

Trust is a relationship normally occurring among humans. In professional contexts, for instance, we trust doctors, lawyers, teachers. It is a form of reliance on someone considered to have adequate level of knowledge and skill (epistemic trust) and moral features (moral trust), such as good intentions and commitment to professional values. It is questionable whether we can meaningfully trust tools, like AI. On some views, the appropriate relationship with tools is not one of trust, but simply one of reliability. (20) It seems trust requires a level of accountability, as to trust someone we need to be ready to feel betrayed by that individual. (21) Thus, to trust AI is metaphorical language for trusting those who design or use it, in the same way as with any other tool. Perhaps we can say that we trust our car, or our laptop, or an AI tool. But that is metaphorical language. Trust is directed at designers or users. As mentioned in the previous sections, they are the ones who are morally responsible and accountable.

### 8 Need for Explanation and Justification

Bjerring and Busch note that if clinicians relying on opaque deep learning systems cannot understand why certain decisions are made, they are unable to communicate this information to the patient, which in turn doesn’t allow the patient to make an informed decision. (22)

On the basis of these arguments, some have argued that patients have a right to refuse diagnostics and treatment planning by AI systems in favour of a human-only alternative. (23) Others have argued in favour of interpretable AI, (24) that is AI constrained so that a human can easily understand it, though this seems impossible with generative AI and LLMs.

Generally, explanation of how something works is valuable, particularly in predicting the conditions under which it will work. However, it is not necessary to justification. Many medical treatments such as aspirin and statins have been employed pragmatically, and without understanding how they benefit. The justification for their use lies in evidence of beneficial effect through clinical trials. We do not ask how a hammer works – we ask whether a human using a hammer did a good job.

Indeed, what matters most is justification, not explanation. What matters to patients is how something will affect their well-being and autonomy, not how it does that. In ethics there is a deep distinction between explanatory or motivating reasons (why someone acted) and normative or justificatory reasons (whether the act was right). Justification in medicine is provided by values that patients endorse (for instance, prolongation of life or improvement of its quality) and validity conferred by adequate scientific research.

### 9 Obsolescence, Dehumanization and Deskilling

One of the great fears around medical AI is that it will make humans obsolete – that is, replace their role in medicine. Their jobs will evaporate and be replaced by AI

A related concern is that humans will become slaves to AI – students will just employ ChatGPT to answer their essay questions; doctors will employ ChatGPT to write reports. This will result in the deskilling of the medical profession.

These concerns might materialise. But if humans can stay “on the loop” by being educated in the science of AI and the relevant ethics, and in engaging patients in both science and ethics, they will play a pivotal role in the future of medicine. One reason to think humans will be kept in the loop is the need for accountability and trust in medicine, which as said above cannot be fulfilled by AI. As long as accountability will be in demand, so will human doctors.

### 10 Catastrophic Dual Use

AI has been used to develop novel lethal drugs (25). AI represents an existential threat. It could be used by a psychopathic or rogue actor to develop some super-lethal biological threat. Or, in a less plausible but not impossible scenario, it could be AI itself developing to such a stage that it becomes self-aware and possess intelligence greater than human beings.

The existential risks warrant regulation, and in many ways. Humans are unfit for the future, including a future of AI. (26) This is a broader problem related to the potential for hams that new technologies allow, and is not specific to AI use in medicine.

## Ethical Reasons to Employ AI, and Benefits of AI

We can now discuss the key ethical benefits from the use of AI in medicine. As with the risks discussed, these benefits are not meant to be exhaustive or mutually exclusive. They indicate our distillation of the key benefits as captured in the literature, as well as through our research.

### 1 Big Data and Better Performance

AI ability to utilize vast amounts of relevant data (Big Data). For example, the Cambridge breast cancer algorithm is based on nearly one million women. (27) In the case of LLMs, clinicians and patients have access to global best expertise. LLMs can potentially bring together knowledge from all of humanity and specifically trained LLMs can produce expert advice rivalling best human doctors. (28) This reduces risks to patients and provides better medical performance in diagnosis and treatment. In this way, it fulfils two core principles of biomedical ethics, namely non-maleficence and beneficence.

### 2 Efficiency, Productivity and Reduction in Human Resources

AI increases speed and power, and is able to replace many human tasks. This increases human productivity, reduces human performance of mundane tasks (eg LLMs could be used to write letters or patient summaries), and can provide greater level expertise in low resource settings, such as the low and middle income countries. For example, medical imaging AI can assist in remote diagnosis of investigations in low income countries. (29)

### 3 Making Values Explicit and Weighing of Values

Every action requires a goal (or a value to be maximized) and a means to achieve that goal. AI is a tool (like a hammer) to achieve human goals. Medicine is a value laden practice. For example, allocation of limited life-saving resources such as ventilators, vaccine, expensive novel therapies, and so on requires balancing or weighing different values such as probability of survival, length of survival, quality of life, responsibility for illness, social utility, dependents, past injustice, age, etc. (30) Medicine is based on a principle of providing interventions which are in the best interests of patients, but this involves evaluation of patient well-being, which is an inherently ethical and philosophical concept surrounded buy much disagreement. Such disagreements are most marked in medical decisions to withhold or withdraw life saving children in children. (31)

The performance of AI must be evaluated and this requires articulation of values to judge performance. AI provides an opportunity to make the values that guide decisions explicit in the assessment, rather than leaving them implicit, unspoken or within the sole purview of medical professionals. For example, once values are made explicit for example relating to coercion in public health, such as mandatory vaccination, it is possible to construct algorithms or decision trees that utilize these values in decision making. (32) Such algorithms are capable of interrogation and revision. Similarly, in organ transplantation, AI requires that values be made more explicit than is currently the case with committees which meet in secret and keep their processes opaque to the public.

Medicine requires thresholds be set for statistical significance, effectiveness and safety – these inherently involve value judgements. (33) Thresholds must similarly be set in AI – for example about the acceptable level of true and false positives and negatives. These decisions involve value judgements, but since these figures need to be made more explicit when AI is assessed, they are liable to scrutiny. For example, in the case of DermAssist, it has been claimed that Google accepted high rates of false-positive results, particularly in dark skinned patients where there was little data, to protect against false negative results, such as failing to diagnose a melanoma. (34) More false positives results in worry to patients, increase in use of limited health resources, with some concerned about a a “tsunami of overdiagnosis. “ (35) Just as where the maximum speed limit is set in different countries is a result of weighing the vectors of economic efficiency, pleasure, convenience, safety, carbon emissions, etc, so too where the thresholds of sensitivity and specificity are set involve complex valuable judgements that warrant public ethical deliberation.

### 4 Precision Medicine and Confidence Limits

Before AI, patients were often presented with large population level data which may or may not be relevant to them. AI has an extraordinary power to provide information about groups, down to smaller and smaller groups. This enables, potentially, different groups to gain more precise information, such as risk information, about them. This is a move towards precision medicine.

In the DermAssist example, the risk predictions were less reliable for dark skinned groups, so confidence is lower. But as AI moves to express more explicit estimates of confidence in prediction for specific groups, this will enable more precise and informed decision making and consent.

### 5 Improvement in Doctor-Patient Relationship

While many worry that AI will undermine the doctor-patient relationship, and that direct-to-consumer AI such as DermAssist will cause patient worry and confusion, AI also has potential to improve the doctor patient-relationship. If doctors are properly educated in AI, they can use these tools to improve performance, reduce mundane tasks and participate with the patient in more personalized medicine. Doctors could also play a vital role in communicating the reliability of different AI tools. It will require in depth knowledge of AI evaluation and performance, and the ability to communicate this meaningfully to patients. Because the programming and evaluation of AI requires making values more explicit, this opens the possibility of more value-based dialogue with patients, for example, over whether they prioritise length or quality of life in cancer care.

### 6 Autonomy, Empowerment and Personalized LLMs

The process of exposing the last few layers of an LLM’s neural network to a specialized text is usually referred to as ‘fine-tuning’., As a result, the model retains its access to global knowledge but its output is influenced by a more specialized training set. (36)

Fine-tuning could be used to produce specialized medical services. For example, LLMs could be trained on information relevant to a particular procedure, such as breast surgery. Patients could have the opportunity to interact and “converse” with such models prior to surgery (“ConsentGPT”). This may produce superior understanding and facilitate better informed consent than consent obtained from time poor, less knowledgeable junior doctors. (37)

Fine tuning could be used to produce personalized models of either doctors or patients. Doctors performing research could train LLMs such as ChatGPT on their existing research so that the model would more accurately reflect their own values, knowledge, contribution and narrative. This has been shown to be superior to the performance of untrained models and could be used as a form of co-creation to generate novel papers, grant applications or presentations. (38) This will require new norms of attribution as well as ensuring sufficient human effort, commitment, originality and contribution to warrant praise. Personalized models could be used by practicing doctors to generate personalized letters, reports and diagnosis or treatment plans.

Personalized models of patients could be produced in a variety of ways: data stored in electronic health records and biobanks; responses to medical questionnaires; value-eliciting choice experiments undertaken by the individual while competent; other text produced by individuals, such as blog posts or other published writings, (39) social media activity(e.g., Facebook ‘likes’), (40) and emails.

These models could be used to create “ethical avatars” of the patient which represents the patient’s values and to some extent their voice. It could be used as a surrogate of the patient in those cases where it is not possible to obtained a patient’s explicit consent, for instance when the patient is unconscious and there is no advance directive. (41)

Since such personalized models would also be fused with the general corpus of human thought and knowledge, the patient could consult them in normative dialogue about which course of action is best for him or her.

Personalized models can also be produced of famous, influential or important people. One model of the famous philosopher Daniel Dennett has been trained on his work. Its responses to novel questions could have been convincingly be attributed to Dennett himself, on the basis of his own responses as predicted by experts. (42) Models of Aristotle, Confucius, Kant, Buddha, Jesus, Peter Singer or Lee Kwon Yiew could be produced and patients could consult them about either prudential (including medical) and moral dilemmas (what we might call a “Moral Guru Model”).

In all these ways, AI offers the prospect of enhancing autonomy, enabling richer, deeper and more informed decision-making and patient empowerment.

### 7 Promoting Justice

AI can be used promote equality, fairness and justice. AI could be used in poorer countries to advance medical decision making and it can also be used to empower patients there. More generally, AI requires both that explicit goals be set and that performance be evaluated. Both of these steps require explicit values and justice can be one of those values. While humans including doctors are “trusted” to make ethical decisions, they are often subject to bias, prejudice and use their own idiosyncratic conceptions of fairness. AI cannot be trusted in this way, but that an ethical advantage since, as argued above, that means those values would need to be made explicit.

### 8 AI Explicit Ethics

Consider the behaviour of cars. Humans will make a decision on how to drive, and how to respond to threats or unexpected events. In contrast, AI is able to respond much faster and to a much wider range of input variables, and must be programmed or at least evaluated in its performance. This requires explicit ethics.

Awad and colleagues (43) in their Moral Machines experiment evaluated over 40 million preferences from 233 different regions on how autonomous vehicles should distribute risks and harms. They found, in order of strength, the following public preferences:

*Human lives over animal lives*
*More lives rather than fewer*
*The young over the elderly*
*Law abiding over law breakers*
*High social status over low social status*
*Healthy weight over those who are overweight*
*Females over males*
*Pedestrians over passengers*
*Preference for the vehicle to continue in its motion, over a vehicle taking evasive action*.

Awad and colleagues argue the 3 strongest preferences provide a basis for public policy (and programming AI).

Public health similarly involves distribution of benefits and burdens, for example, through policies related to restriction of liberty and coercion, such as quarantine, isolation, lockdown and mandatory vaccinations. However, questions of justice are not restricted to public health in medicine. Studies have been conducted on the allocation of ICU beds, ventilators and vaccines in the pandemic, involving evaluation of chance of survival, length of survival, quality of life, age, social utility, etc. (44) Ethical Algorithms have been constructed for vaccination and allocation of ventilators. (45) Box 1 illustrates how widespread dilemmas involving distribution of harms and benefits are.

**Fig 1:**
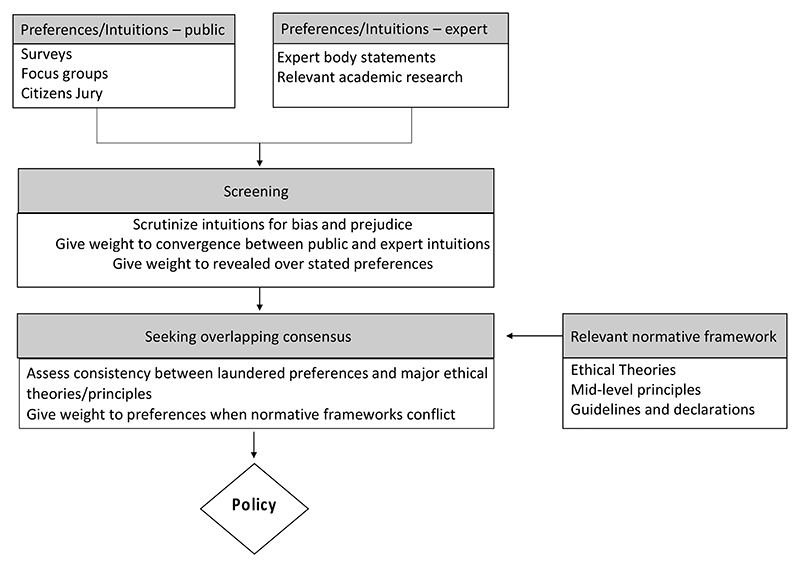
Collective Reflective Equilibrium

How should such ethical decisions be made about the programming or evaluation of AI? Given the observed diversity of ethical risks that arise from the use of AI in medicine, a general approach to making progress against them requires fluidity and adaptability. Fig 1 and Box 2 outline the basic concepts and approaches. We argue that an effective approach needs to contain a diversity of conceptual and ethical tools to draw upon for tackling the varied risks (as in Box 2), but the application of such tools needs to be grounded in an overarching methodology (such as Collective Reflective Equilibrium, as below and in Fig 1). The suggestions below are a start on how we might start assembling such a flexible toolkit.

**Collective Reflective Equilibrium** is an approach which aims to bring both public preferences and ethical theories/concepts/principles into maximum coherence (46) – see Fig 1. It would be a mistake for such decisions to blindly follow people’s preferences. Public views on moral questions can be prejudiced, biased or deeply mistaken, such as when there is low support for organ donation, despite a shortage of organ donation being rightly seen as a problem to be overcome. See Fig 1 below. (47)

## Conclusion

AI is, in one way, just another human tool. It requires humans to use it well. But it also makes ethics unavoidable – we must now face the values which drive our choices and which ground the evaluation of our actions.

Arguably, AI is the greatest experiment humans have ever undertaken. It will radically transform medicine. Whether we master it, and reap its enormous potential, or become slaves to it, and undermine our very humanity and that of our patients, is up to us.

## Figures and Tables

**Table 1:** Summary of Ethical Risks of AI in Medicine

Effectiveness, Reliability and Evaluation
Justice, Inequality, Bias, Discrimination, and Fairness
Privacy and Confidentiality
Machine Paternalism and Respect for Autonomy
Accommodating Value Pluralism and Disagreement
Responsibility Gaps and Accountability
Trust
Need for Explanation and Justification
Obsolescence, Dehumanization and Deskilling
Catastrophic Dual Use

**Table 2:** Summary box of recommendations for use of AI in embryo selection.

**ï**	Use of replicable, interpretable ML tools and data
**ï**	Well designed and conducted RCTs
**ï**	Post implementation surveillance
**ï**	Regulatory oversight requiring interpretable AI whenever possible
**ï**	Funding for public institutions to transparently develop and evaluate ML models, and open access to code used in models
**ï**	Procedures for maintaining security of patient/embryo data while permitting etdical data sharing
**ï**	Fully informed consent to use A
**ï**	Inclusion of patient values into AI programs where possible
**ï**	Training for clinicians to understand AI models and explain them to patients

AI, artificial intelligence; ML, machine learning; RCT, randomized controlled trial.

**Table 3:** Summary of Ethical Reasons to Employ AI

Big Data and Better Performance Efficiency, Productivity and Reduction in Human Resources Making Values Explicit and Weighing of Values Precision Medicine and Confidence Limits Improvement in doctor-patient relationship Autonomy, Empowerment and Personalized LLMs Promoting Justice AI Explicit Ethics

## Box 1 Examples of Dilemmas about Justice and Distribution of Benefits and Harms

Distribution of medical resources/limitation to treatment

Organ allocation for transplantation

Infectious disease - quarantine/lockdown/mandatory vaccination

Antimicrobial resistance

Road, food, environmental and other safety

Autonomous vehicles

Carbon emissions/climate change

War/drones
